# Factor structure and psychometric properties of the affective lability scale-short form in Chinese adolescents

**DOI:** 10.3389/fpsyt.2022.881541

**Published:** 2022-11-17

**Authors:** Shuyin Xu, Yafei Chen, Yunjing Li, Siqi Yang, Yimei Lu, Liang Li, Mei Huang, Mohan Ma, Wenwen Ou, Guanyi Lv, Xiaotian Zhao, Yaqi Qin, Yumeng Ju, Yan Zhang, Lingjiang Li

**Affiliations:** ^1^Department of Psychiatry and National Clinical Research Center for Mental Disorders, The Second Xiangya Hospital of Central South University, Changsha, Hunan, China; ^2^Xiangya Medical School, The Second Xiangya Hospital, Central South University, Changsha, Hunan, China

**Keywords:** affective lability scale-short form, adolescents, reliability, validity, depression

## Abstract

**Objective:**

Previous studies on the reliability and validity of the Affective Lability Scale short-form (ALS-SF) have only been evaluated in adults, which may not be able to generalize to the adolescent population. We aimed to examine the factor structure, the reliability and validity of ALS-SF among Chinese adolescents and construct an adolescent form of ALS (ALS-AF).

**Methods:**

A total of 1,439 middle school students were investigated with a broad survey including ALS-SF, Patient Health Questionnaire-9 (PHQ-9), Generalized Anxiety Disorder 7-item (GAD-7), Connor-Davidson Resilience Scale 10-item (CD-RISC-10) and non-suicidal self-injury (NSSI) behavior self-report. Exploratory factor analysis (EFA) and confirmatory factor analysis (CFA) were conducted to investigate the structural validity of ALS-SF and construct ALS-AF. Cronbach's α was used to assess the internal consistency and reliability of the scale. Factor loading, Average Variance Extracted (AVE) and Composite Reliability (CR) were applied to measure the convergent validity and divergent validity. Besides, Correlation and regression analyses were used to explore the relationship between affective lability and depression, anxiety, NSSI and resilience.

**Results:**

Factor analysis failed to support the original three-factor model of 18-item ALS-SF and confirmed the three-factor model of 15-item ALS-AF. The ALS-AF showed good internal consistency as well as strong convergent and discriminative validity. Besides, ALS-AF was positively correlated with PHQ-9, GAD-7 and self-harm, and was negatively associated with resilience.

**Conclusion:**

Our study shows that the ALS-AF has good reliability and validity for testing affective lability in Chinese adolescents.

## Introduction

Adolescence is the beginning development of more complex behavior and cognitive processes such as emotion regulation and decision making (1). This period is vulnerable to problems in the regulation of affect and behavior (2, 3). The affective lability, which refers to abnormal and frequent shifts in emotional state over a short space of time, is a known feature during the developmental phase of adolescence (4). The affective instability in adolescents has been shown to be related to later mental disorders, including general anxiety disorder, major depressive disorder, bipolar disorder and borderline personality disorders (1, 5–7). In addition, affective instability has been found to be an essential risk factor for suicide attempts and self-harm among youths (8). Characterizing the affective lability in adolescents may provide meaningful information on their vulnerability to mental disorders. Thus, the evaluation of affective lability is warranted in adolescents.

At present, the most widely used scale for assessing emotional stability in clinics is the 18-item Affective Lability Scale short-form (ALS-SF), which was developed by Oliver and Simon on the basis of ALS-54 (9). The scale measures the tendency of individuals to frequently shift between different emotional states, including calm, anxiety, depression, elation and anger. Three factors were identified in the ALS-SF (9): anxiety/depression (AD), depression/elation (DE) and anger (Ang). The reliability and validity of the scale have been established in healthy samples (9–11) and clinical groups such as personality disorders (12), bipolar disorder (13) and attention deficit hyperactivity disorder (ADHD) (14) (see Table 1 for details). However, no studies have applied the scale in China, and most of the subjects enrolled were adults. There is currently no research on the validity and reliability of the ALS-SF in adolescents. Adolescents have unique psychological characteristics as they assert their independence and adopt more impulsive behaviors. Mood swings and anxiety also significantly increased at puberty (15, 16). Hence, the emotional stability characteristics in adolescents are quite different from adults.

**Table 1:** Psychometric property studies of the ALS-SF.

**Participants**	***N***	**Age**	**AD**	**Ang**	**DE**	***χ^2^*/df**	**RMSEA**	**CFI**	**TLI**	**SRMR**	**Country**	**Year**
Pregnant and postpartum women	113	29.17 ± 4.9	0.80	0.91	0.71	2.11	0.1	0.88	0.86	-	Canada	2019
General population	494	31.73 ± 12.6	0.91	0.89	0.89	2.78	0.061	0.99	-	0.055	Italy	2018
ADHD Healthy control	187 48	33.66 ± 12.0	0.86	0.86	0.87	-	0.077	0.91	-	0.053	Switzerland France	2017
Bipolar disorder First-degree relatives Healthy control	422 201 307	42 ± 13 52 ± 16 39 ± 12	0.88 0.88 0.86	0.81 0.77 0.79	0.85 0.87 0.83	3.99 1.67 2.43	0.084 0.058 0.062	0.96 0.99 0.98	0.95 0.98 0.97	- - -	Norway France	2015
Cluster B personality disorders Other personality disorders Healthy control	236 180 164	35.08 ± 10.4 36.24 ± 12.1 30.04 ± 9.2	0.82	0.84	0.78	3.87	0.07	0.95	-	0.07	America	2010
Undergraduates	372	19.99 ± 1.54	0.87	0.82	0.81	2.51	0.06	0.92	-	-	America	2004

ALS-SF, affective lability scale-short form; ADHD, attention deficit hyperactivity disorder; AD, anxiety/depression; Ang, anger; DE, depression/elation. χ^2^, Chi-square; df, degrees of freedom; RMSEA, root-mean-square-error of approximation; SRMR, standardized root-mean residual; CFI, comparative fitness index; TLI, Tucker–Lewis indices.

According to the principles of psychometrics, it is necessary to test the reliability and validity when a scale is applied to groups with different characteristics. As previous models may not fit the adolescent population very well, our current study aimed to test the reliability and validity of ALS-SF and construct an adolescent form of ALS (ALS-AF) in a large non-clinical adolescent sample. In addition, given the high correlation between ALS and mental health issues, we also investigated the association of affective lability with depression, anxiety, self-harm and resilience.

## Methods

### Participants and procedures

The survey was conducted using a convenient sampling procedure from February 12th to May 12th, 2021. The participants of this study were recruited from 6 districts in Changsha, Hunan Province, China. Within each district, samples were from 2 classes from 1 to 3 middle/high schools. A total of 1,501 questionnaires were distributed, 1,439 valid questionnaires were encoded, and the response rate was 95.8%. The Ethics Committee of the Second Xiangya Hospital of Central South University approved this study.

The inclusion criteria were: (1) 12–19 years old; (2) with parents gave consent to participate in the study; (3) reported without severe physical disease (e.g., heart, lung, liver, or kidney disease) or abnormal physical and mental development (pygmyism, mental retardation, etc.). Data were collected using paper versions of a self-administered questionnaire, and all answers were anonymous. Before the survey, a well-trained psychiatrist or psychologist would inform the participants about the objectives and processes of the study. Written informed consent was obtained from all the participants and their parents. All the questionnaires were manually checked. Questionnaires that did not meet the inclusion criteria and had obvious filling errors were eliminated.

### Measures

#### Demographic characteristics

Social demographic data included gender, age, residence (urban/rural), graduating student (yes /no), only child in family (yes/no), parental education level and family income (CNY).

#### Affective lability

Affective lability was assessed using Affective Lability Scale short-form (ASL-SF), which was authorized by Professor Jeffrey S. Simons from the University of South Dakota. One psychiatrist translated the questionnaire into Chinese (YJ) and another psychiatrist (FS) who had not seen the original version back-translated into English to ensure the Chinese version was equivalent to the English version. Then a senior expert (YZ) proofread and modified the scale. Next, the researcher (SX) asked 10 teenage students about their understanding and suggestions of the scale and revisions were made after summarizing their opinions. The ASL-SF consists of 18 items which are divided into three subscales: anxiety/depression (item 1, 3, 5, 6, 7), depression/elation (item 2, 10, 12, 13, 15, 16, 17, 18), and anger (item 4, 8, 9, 11, 14). The items were scored with the Likert scale of 0–3 points (0 = “very uncharacteristic of me” to 3 = “very characteristic of me”). A total score (ranged from 0 to 54 points) was obtained by summing the scores for each of the 18 items. Many previous studies have confirmed the strong psychometric properties of ALS-SF in the adult population (Table 1).

#### Depression and anxiety

Depression levels were assessed by the Chinese version of the Patient Health Questionnaire-9 (PHQ-9), which mainly asks about the frequency of depressive symptoms occur during the past 2 weeks. The scale has shown high reliability (Cronbach's α = 0.81–0.88) among high school teenagers (17, 18). Anxiety levels were examined by the Chinese version of the Generalized Anxiety Disorder 7-item (GAD-7), which also demonstrated high internal consistency (Cronbach's α = 0.85–0.91) in teenage students (19, 20). Each question in PHQ-9 and GAD-7 was scored with the Likert scale of 0–3 points (0 = “not at all” to 3 = “nearly every day”).

#### Resilience

Resilience was assessed using the 10-item Connor-Davidson Resilience Scale (CD-RISC-10), asking about how much a statement applies to them during the last month. The scale has shown high reliability (Cronbach's α = 0.88–0.90) among Chinese high school teenagers (21, 22). Each question in CD-RISC-10 was rated from 0 to 4 (0 = “not true at all” to 4 = “true nearly all of the time”), with higher scores suggesting greater resilience.

#### Self-harm

Participants were asked about non-suicidal self-injury (NSSI) behavior using the question from the inventory of statements about self-injury (ISAS): “Please estimate the number of times in your life you have intentionally (i.e., on purpose) performed each type of non-suicidal self-harm (e.g., 0, 10, 100, 500).” The number of times they had performed self-harm behavior was summed to assess self-harm frequency.

### Statistical analysis

Electronic Data Capture System is used to double-enter the data (SY, YC, LL, YL, MH, XL, SY, JL, YC). All data were then checked by other investigators (YJ, SX) to identify any errors or discrepancies and then finally imported into statistical software. Analyses were conducted in SPSS 20.0 and Mplus 8.0 software. For sociodemographics, continuous variables were presented as appropriate for median and interquartile range or mean and standard deviation. Categorical variables were presented as frequency and percentages in each category. Pearson correlation analysis and multiple linear regression analysis were used to explore the relationship between affective lability and depression, anxiety, NSSI and resilience.

Cronbach's α was used to assess the internal consistency and reliability of the scale. The Cronbach's α > 0.7 indicates high internal consistency (23). A combination of exploratory factor analysis (EFA) and confirmatory factor analysis (CFA) was used to investigate the structural validity of ALS-SF. The data sets were randomly divided into two halves, one half used for EFA and the other half used for CFA. EFA was conducted using principal axis factor (PFA) and maximum variance rotation to examine the validity and factor structure of ALS-SF (24). The appropriateness of factor analysis was determined using the Kaiser-Meyer-Olkin (KMO) and Bartlett's test of sphericity measure. The KMO > 0.5 and Bartlett spherical test (*P* < 0.05) indicate that data is suitable for factor analysis. The cut-off for the factor loading of each item was set at 0.5. After model fitting by EFA, CFA was used to examine the model structure. The χ^2^/df, root mean square error of approval (RMSEA), standardized root mean square residual (SMSR), comparative fit index (CFI) and Tucker Lewis index (TLI) were used to evaluate the goodness of fit of the model. χ^2^/df < 5, RMSEA < 0.08, SMSR < 0.06, CFI > 0.90, TLI > 0.90 indicate a good model fit (25, 26).

Convergent validity and divergent validity were measured by factor loading, Average Variance Extracted (AVE) and Composite Reliability (CR). The correlation between items and factors measures factor loading. AVE is measured by the level of variance explained by the construct vs. the level due to measurement error. CR is measured by the consistency of items within the factors. Factor loading > 0.60, AVE > 0.50 and CR > 0.60 suggest good convergent validity. The square root of the AVE for each factor higher than the correlation with any other factor indicates good divergent validity (27–29).

## Results

### Sample characteristics

Among 1,439 participants, 44% were male, and 56% were female, with a mean age of 14.93 ± 1.78. The mean PHQ score was 8.69 ± 6.10; GAD was 6.54 ± 5.38; CD-RISC-10 was 21.01 ± 8.90. Table 2 reports the sociodemographic characteristics of the sample.

**Table 2:** Sample characteristics (*n* = 1,439).

**Characteristics**	**Variable**	**Percent%**
Gender	Male Female	44.0 56.0
Age (years)	12–14 15–17 18–19	52.6 33.4 14.0
Residence	Urban Rural	64.4 34.6
Graduating student	YES NO	42.1 57.9
One-child family	YES NO	33.1 66.9
Parent's educational level (Father/Mother)	Middle school or below High school or above	49.7/57.0 50.3/43.0
Family monthly income level (CNY)	< 5,000 5,000–10,000 >10,000	39.5 38.2 22.3

### Results of reliability and validity analyses

The result of EFA showed that the ALS-SF was suitable for factor analysis (KMO = 0.941; Bartlett's test = 6787.54, *P* < 0.001). PAF in EFA yielded three factors, explaining 53.47% of the total variance. Since the factor loading of item 2, item 4, and item 10 was <0.5, they were deleted from the factors. The factor loadings of all the other items were between 0.523 and 0.774. In addition, item 8 loaded highly on both factor 1 and 3 (factor loading = 0.507 for factor 1, factor loading = 0.570 for factor 3). Since the content of item 8 is closer to factor 3, we grouped it into factor 3. Based on the content of the items and the original three-factor model structure, the identified three factors were named anxiety/depression (AD), anger (Ang) and depression/elation (DE), respectively. Eventually, an adolescent form of ALS (ALS-AF) containing 15-item with a three-factor structure was constructed.

To further determine the optimal factor structure of the scale, we perform CFA on ALS-AF with both current and original three-factor model and on ALS-SF with the original three-factor model. Results of fit indexes for the three-competition model are shown in Table 3. The goodness-of-fit of the three models showed that the ALS-AF three-factor model had the best model fit (*P* < 0.01, RMSEA = 0.078, SRMR = 0.054, CFI = 0.93, TLI = 0.92).

**Table 3:** Fit indices for the competing factor models (*n* = 744).

	**Model**	***χ^2^***	**df**	***χ^2^*/df**	**RMSEA (90%CI)**	**SRMR**	**CFI**	**TLI**
ALS-AF	Three-factor model	485.12	87	5.58	0.078 (0.072–0.085)	0.054	0.932	0.919
	Original three-factor model	585.83	87	6.73	0.088 (0.081–0.095)	0.061	0.915	0.898
ALS-SF	Original three-factor model	907.52	132	6.88	0.088 (0.083–0.094)	0.061	0.889	0.871

ALS-AF, affective lability scale-adolescent form; ALS-SF, affective lability scale-short form; χ^2^, Chi-square; df, degrees of freedom; RMSEA, root-mean-square-error of approximation; SRMR, standardized root-mean residual; CFI, comparative fitness index; TLI, Tucker–Lewis indices.

Table 4 shows the reliability and convergent validity of the ALS-AF three-factor model. Cronbach's α achieved 0.925 and the factor loading all passed 0.6, suggesting that the scale has good reliability and validity. Table 5 shows the result of discriminative validity. The square root of AVE for each factor is greater than the correlation coefficient between the factor and other factors, indicating that the scale has good discriminative validity.

**Table 4:** Mean ± SD, reliability and convergent validity for the ALS-AF (*n* = 1,439).

**Items of the scales**	**Mean ±SD**	**Factor** ** loading**	**AVE**	**CR**	**Cronbach' α**
**Anxiety/Depression**			0.597	0.880	0.874
1. I feel just as relaxed... and then dizzy	0.92 ± 0.88	0.714^**^			
2. I can be feeling OK and then... jittery and nervous	1.10 ± 0.92	0.793^**^			
3. I feel nervous... and then... very sad and down	1.12 ± 0.97	0.874^**^			
4. I go from feeling extremely anxious... to... down	1.07 ± 0.96	0.845^**^			
5. I shift back and forth from... calm to... nervous	1.09 ± 0.95	0.609^**^			
**Anger**			0.606	0.821	0.812
6. I feel perfectly calm... and then... makes me furious	1.09 ± 1.01	0.809^**^			
7. I will be felling OK but then I... get mad	0.91 ± 0.96	0.839^**^			
8. I am so mad... and other times.... I get so mad	0.90 ± 0.96	0.679^**^			
**Depression/Elation**			0.481	0.866	0.865
9. I switch... between... energetic and... little energy	1.10 ± 0.94	0.672^**^			
10. I feel absolutely wonderful... but soon... the same	1.21 ± 0.93	0.612^**^			
11. I'm so mad that my heart starts pounding	0.93 ± 0.90	0.627^**^			
12. I shift... between unproductive and... productive	1.00 ± 0.90	0.722^**^			
13. I feel extremely energetic... then... little energy	1.13 ± 1.00	0.757^**^			
14. I have more energy... then... the same...as everyone	1.03 ± 0.90	0.757^**^			
15. I'm doing everything... slow but then... I'm no more	1.14 ± 0.94	0.691^**^			

SD, standard deviation; ALS-AF, affective lability scale-adolescent form; AVE, average variance extracted; CR, composite reliability.

** p < 0.01.

**Table 5:** Discriminatory validity for the ALS-AF (*n* = 1,439).

	**AD**	**Ang**	**DE**
AD	0.773		
Ang	0.600^**^	0.778	
DE	0.634^**^	0.690^**^	0.694

ALS-AF, affective lability scale-adolescent form; AD, anxiety/depression; Ang, anger; DE, depression/elation.

** p < 0.01.

### Relationship between ALS and demographic and psychological characteristics

The mean ALS-AF score of the whole sample is 1.05 ± 0.65. There was a significant difference in the ALS-AF score between female (1.15 ± 0.61) and male students (0.91 ± 0.68) (*P* < 0.001), non-graduates (1.01 ± 0.69) (*P* < 0.001) and graduating students (1.08 ± 0.83), students live in urban areas (1.08 ± 0.67) and students live in rural areas (1.01 ± 0.61) (*P* < 0.05). No significant difference was observed between only child in family and child who have siblings. Besides, no correlation has been detected between students' age, parents' educational level or family income with ALS-AF score.

Pearson correlation analyses showed a high correlation between the total score of ALS-AF and ALS-SF. Moreover, the correlation between the two ALSs with PHQ-9, GAD-7, NSSI and CD-RISC-10, respectively, were also highly similar. The ALS-AF positively correlated with PHQ-9, GAD-7 and NSSI, and is negatively correlated with CD-RISC-10 (Table 6). The variance inflation factor (VIF) of the three independent variables is 2.419, 2.688, and 2.205, respectively, indicating that there is no significant multicollinearity (VIF < 5) between the variables. Results of multiple linear regression analyses showed AD and Ang positively correlated with PHQ-9 and GAD-7, while DE negatively correlated with PHQ-9 and GAD-7. Similarly, AD and Ang were related to NSSI positively, and DE correlated with NSSI negatively. Besides, AD was positively associated with CD-RISC-10, and DE was negatively associated with CD-RISC-10 (Table 7).

**Table 6:** Correlation between affective lability and depression, anxiety, self-harm and resilience (*n* = 1,439).

	**AD**	**Ang**	**DE**	**ALS-AF**	**ALS-SF**	**PHQ**	**GAD**	**NSSI**	**RISC**
AD	1								
Ang	0.738^**^	1							
DE	0.667^**^	0.707^**^	1						
ALS-AF	0.877^**^	0.882^**^	0.904^**^	1					
ALS-SF	0.878^**^	0.891^**^	0.913^**^	0.993^**^	1				
PHQ	0.574^**^	0.472^**^	0.397^**^	0.525^**^	0.525^**^	1			
GAD	0.615^**^	0.501^**^	0.397^**^	0.555^**^	0.548^**^	0.791^**^	1		
NSSI	0.298^**^	0.270^**^	0.174^**^	0.267^**^	0.265^**^	0.349^**^	0.315^**^	1	
RISC	−0.344^**^	−0.223^**^	−0.153^**^	−0.265^**^	−0.257^**^	−0.411^**^	−0.397^**^	−0.242^**^	1

AD, anxiety/depression; Ang, anger; DE, depression/elation; ALS-AF, Affective Lability Scale-adolescent form; ALS-SF, Affective Lability Scale-short form; PHQ, Patient Health Questionnair-9 item; GAD, Generalized Anxiety Disorder-7 item; NSSI, non-suicidal self-injury; RISC, Resilience Scale.

** p < 0.01.

**Table 7:** Multiple linear regression analyses on the effect AD, and DE on PHQ, GAD, NSSI and RISC.

**Variables**		**β**	***t***	***p***	***R* ^2^**	***F***	***P***
PHQ	AD	0.800	15.03	0.000^**^	0.335	240.97	0.000^**^
	Ang	0.195	3.24	0.001^**^			
	DE	−0.023	−0.626	0.532			
GAD	AD	3.966	17.56	0.000^**^	0.386	301.15	0.000^**^
	Ang	0.632	4.13	0.000^**^			
	DE	−0.554	−2.58	0.010^*^			
NSSI	AD	0.888	6.47	0.000^**^	0.100	52.99	0.000^**^
	Ang	0.363	3.90	0.000^**^			
	DE	−0.380	−2.90	0.004^*^			
RISC	AD	−5.082	−11.43	0.000^**^	0.129	70.97	0.000^**^
	Ang	0.021	0.07	0.945			
	DE	1.588	3.75	0.000^**^			

AD, anxiety/depression; Ang, anger; DE, depression/elation; PHQ, Patient Health Questionnaire-9 item; GAD, Generalized Anxiety Disorder-7 item; NSSI, non-suicidal self-injury; RISC, Resilience Scale.

* p < 0.05;

** p < 0.01.

## Discussion

This is the first study to examine the psychometric properties of the ALS-SF among Chinese adolescents. EFA and CFA did not strongly support the three-factor model of ALS-SF developed by Oliver and Simons in our adolescent sample (9). On the other hand, the three-factor model of ALS-AF showed good validity and reliability. In total, our results indicate that the ALS-AF can produce valid and reliable assessments of affective lability among adolescents.

Our results showed that the model fitting indexes of ALS-AF were better than that of the ALS-SF version. Except for χ^2^/df, which is increased due to the large sample size. Compared with the original ALS-SF model, the factor structure of ALS-AF remained similar, with three items been deleted. Item 4 (“I... control my temper... to not being able to control it”) was removed from the Ang factor. The items in the Ang factor are about emotion generation, whereas the item 4 is more related to emotion control. We interpret this may be the reason of the exclusion of item 4 from Ang. Item 2 (“I have very little energy and then … the same”) and item 10 (“I can think clearly … then … difficulty concentrating”) had been deleted from the DE factor. The item 2 is more related to the lack of energy and may not reflect the elation trait in affective lability. And the item 10 is associated with the fluctuations in concentration; therefore, it may not be relevant to depression/elation in affective lability. Besides, item 14 (“I'm so mad that my heart starts pounding…”) originally belonging to the Ang factor is assigned to the DE factor. In general, our findings suggest that the three-factor model of ALS-AF was valid and reasonable among the teenage population.

Our results also demonstrated a higher level of affective lability in girls than boys, which is inconsistent with previous studies (9, 10). Such inconsistency might be partly owing to the difference in participants. Some studies have shown that adolescent girls have higher interpersonal stress exposure and stress reactivity than adolescent boys (30, 31), and girls are more vulnerable to the stress-sensitive effect during adolescent development (32, 33). Besides, our results revealed that the affective lability in graduating students was higher than that in non-graduates, indicating that the graduating students were more likely to have fluctuations in mood under the pressure of entrance examination. Lastly, the location of residence also affects affective lability, with higher ALS presented in urban students. Such findings may be related to the tremendous competitive pressure in urban areas (34).

In addition, correlation analysis showed that affective lability was positively correlated with depression, anxiety and the frequency of NSSI, and was negatively correlated with resilience. Further regression indicated that AD and Ang positively predicted depression, anxiety and NSSI, whereas DE negatively predicted depression, anxiety and NSSI. In addition, AD is negatively related to resilience and DE is positively associated with resilience. These results suggest that AD and Ang might be risk factors for depression and anxiety symptoms, and DE may be a protective factor in mental health outcomes. The previous study examined affective lability in pregnant and postpartum women; the AD factor also exhibited a positive correlation with depressive and anxiety symptoms, whereas the DE factor had little effect on depression and anxiety (11). The DE factor's positive impact on resilience may be explained by the unique psychological characteristics of adolescents. Adolescence is characterized by declines in positive effect and non-linear patterns of alternating decreases and increases in negative affect (33). During such a rapid development of psychological, physiological and social functions, DE may play a positive role in adaptability and stress resistance.

Our study has several strengths and limitations that merit consideration. Firstly, the sample size of our study is relatively large, which may increase the stability of the results. Secondly, unlike other psychometric property studies of ALS-SF (10–14), we performed a cross-validation test to evaluate the model structure, further increasing the reliability of the three-factor model. Nevertheless, there were some limitations in our study. First, the students we recruited were non-clinical samples, which may have limited generalization to other clinical populations. Besides, our study was conducted at localized district; therefore, the results may have limited generalizability to other sites. Second, the samples were recruited by convenient sampling, there could also be a response bias (e.g., individuals who were more depressed were less motivated to participate in the survey). Random sampling methods could be applied in future studies to minimize this bias. Third, we did not perform test-retest reliability analysis, which prevents us from knowing the stability and consistency of the scale over time. Future research to examine the test-retest reliability of ALS items are warranted to fully validate the scale.

## Conclusion

Our results indicate that the Chinese version of the ALS-AF can produce valid and reliable assessments of affective lability in non-clinical teenage students. Furthermore, the three-factor model has the highest reliability describing affective lability. Future research is warranted to investigate the reliability and validity of ALS in clinical samples of adolescents, so as to facilitate the further promotion and application of the scale in clinical and scientific research.

## Data availability statement

The raw data supporting the conclusions of this article will be made available by the authors, without undue reservation.

## Ethics statement

The studies involving human participants were reviewed and approved by the Ethics Committee of the Second Xiangya Hospital of Central South University. Written informed consent to participate in this study was provided by the participants' legal guardian/next of kin.

## Author contributions

Conceptualization and writing-original draft: YJ and SX. Writing-review and editing: SX, YJ, YZ, and LinL. Data curation: YC, YLi, SY, YLu, LiaL, MH, MM, WO, GL, XZ, and YQ. All authors contributed to the article and approved the submitted version.

## Funding

This work was supported by the National Key Research and Development Program of China (2021ZD0202000), the National Natural Science Foundation of China (82101612 to YJ), the science and technology innovation Program of Hunan Province (2021RC2040 to YJ), the Natural Science Foundation of Hunan Province, China (2022JJ40692 to YJ), and the National Key Research and Development Program of China (2019YFA0706200 to YZ). YJ work was supported by the Postdoctoral Programme of Central South University. The authors would gratefully acknowledge financial support from the above affiliations. The sponsors had no role in the study design, data collection and analysis, and interpretation of the results and report writing.

## Conflict of interest

The authors declare that the research was conducted in the absence of any commercial or financial relationships that could be construed as a potential conflict of interest.

## Publisher's note

All claims expressed in this article are solely those of the authors and do not necessarily represent those of their affiliated organizations, or those of the publisher, the editors and the reviewers. Any product that may be evaluated in this article, or claim that may be made by its manufacturer, is not guaranteed or endorsed by the publisher.
